# A Blood Meal Enhances Innexin mRNA Expression in the Midgut, Malpighian Tubules, and Ovaries of the Yellow Fever Mosquito *Aedes aegypti*

**DOI:** 10.3390/insects8040122

**Published:** 2017-11-06

**Authors:** Travis L. Calkins, Peter M. Piermarini

**Affiliations:** Department of Entomology, Ohio Agricultural Research and Development Center, The Ohio State University, Wooster, OH 44691, USA; tcalkins@tamu.edu

**Keywords:** innexin, *Aedes aegypti*, qPCR, RNAi, blood feeding

## Abstract

Mosquitoes are vectors of pathogens that cause diseases of medical and veterinary importance. Female mosquitoes transmit these pathogens while taking a blood meal, which most species require to produce eggs. The period after a blood meal is a time of extreme physiological change that requires rapid coordination of specific tissues. Gap junctions (GJ) are intercellular channels that aid in the coordination of cells within tissues via the direct transfer of certain small molecules and ions between cells. Evolutionarily distinct groups of proteins form the gap junctions of vertebrate and invertebrate animals (connexins and innexins, respectively). *Aedes aegypti* mosquitoes possess six genes encoding innexins: *inx1*, *inx2*, *inx3*, *inx4*, *inx7*, and *inx8*. The goal of this study was to identify potential roles of innexins in the physiology of mosquitoes after a blood meal by using qPCR to quantify their mRNA expression in adult females at 3 h and 24 h post-blood meal (PBM) relative to non-blood-fed controls. We found that at 24 h PBM, expression levels of *inx2*, *inx3*, and *inx4* mRNAs increased; *inx2* was the most highly upregulated innexin in key tissues associated with blood-meal digestion and egg production (i.e., the midgut and ovaries, respectively). However, knocking down *inx2* mRNA levels by over 75% via RNA interference had no significant effect on fecundity. Altogether, our results suggest that a blood meal influences the molecular expression of innexins in mosquitoes, but their specific physiological roles remain to be elucidated.

## 1. Introduction

Mosquitoes transmit many pathogens that cause deadly and debilitating diseases. The yellow fever mosquito, *Aedes aegypti*, is the primary vector of chikungunya, dengue, yellow fever, and Zika viruses. These pathogens are transmitted when an infected female mosquito feeds on a vertebrate host; females require vertebrate blood in order to produce eggs. Following the consumption of a blood meal, the mosquito goes through a complex series of physiological changes that require the rapid endocrine coordination of multiple tissues [1]. Intercellular channels known as gap junctions allow for endocrine signals to be rapidly shared among adjacent cells within a tissue by mediating the direct transport of ions, small molecules, and second messengers between cells [2].

Gap junctions are comprised of proteins known as connexins in vertebrates and innexins in invertebrates; they are evolutionarily distinct proteins that have convergently evolved to share similar structure and function [3,4]. Some of the broad functional roles that innexin and connexin channels contribute to include development, immune responses, and reproduction [5,6,7,8,9,10]. However, the physiological roles of gap junctions in mosquitoes have only recently begun to emerge.

In *Ae. aegypti*, innexins are (1) expressed in a life stage- and tissue-dependent manner; (2) implicated in the physiology of renal (Malpighian) tubules and the diuretic capacity of adult female mosquitoes; (3) critical for the survival of larval and adult female mosquitoes [11,12,13,14]. In *Anopheles gambiae*, *inx4* (a.k.a. zero population growth) is required for proper male gonad formation, while *inx1* is necessary for immune response to invading *Plasmodium* parasites [8,15]. As such, gap junctions appear to play important roles in mosquito biology.

## 2. Methods

### 2.1. Mosquito Rearing

Eggs of *Ae. aegypti* were obtained through the Malaria Research and Reference Reagent Resource Center (MR4) as part of the BEI Resources Repository (Liverpool strain; LVP-IB12 F19, deposited by M.Q. Benedict). Mosquitoes were reared to adults and the colony was maintained as described in Piermarini et al. [16]. In brief, larvae and adults were held in an environmental chamber set at 28 °C and 80% relative humidity with a 12 h:12 h light:dark cycle. Larvae were reared in plastic trays in distilled water and fed ground TetraMin tropical fish flakes (Tetra Spectrum Brands, Blacksburg, VA, USA). Adults were housed in 0.5 m^3^ cages with access to 10% sucrose, and fed heparinized rabbit blood (Hemostat Laboratories, Dixon, CA, USA) through a membrane feeder (Hemotek, Blackburn, UK) to produce eggs.

### 2.2. Blood Feeding

Mosquitoes were blood-fed three to seven days post eclosion. Twenty-four hours prior to blood feeding, sucrose was removed from the mosquito cages. Mosquitoes were then offered heparinized rabbit blood (Hemostat Laboratories) for 60 min using an artificial membrane feeding system (Hemotek). Control mosquitoes (non-blood-fed) were treated similarly and provided access to 10% sucrose instead of blood for 60 min. After blood feeding, both groups were provided with 10% sucrose. Mosquitoes for tissue level qPCR analysis were dissected at either 3 h or 24 h post-blood meal.

### 2.3. Dissection, RNA Extraction, and cDNA Synthesis

Dissections, RNA extraction, and cDNA synthesis were performed as described in Calkins et al. [11]. In brief, mosquitoes were anesthetized on ice before being dissected in mosquito Ringer solution (consisting of 150 mM NaCl, 3.4 mM KCl, 1.7 mM CaCl_2_, 1.8 mM NaHCO_3_, 1 mM MgCl_2_, 5 mM Glucose, 25 mM HEPES; pH 7.1). Tissues were isolated, transferred to 1.5-ml micro-centrifuge tubes (Thermo Fisher Scientific, Waltham, MA, USA), and preserved in TRIzol^®^ reagent at −80 °C until utilized in RNA isolation. Total RNA was isolated using the method of Chomczynski and Sacchi [17] and quantified using a NanoDrop 2000 spectrophotometer (Thermo Scientific, Wilmington, DE, USA). cDNA libraries were synthesized using 4 µg of total RNA and the GoScript^TM^ Reverse Transcriptase system with random primers (Promega, Madison, WI, USA), following manufacturer’s protocols. cDNA libraries were stored at −20 °C until needed for qPCR.

### 2.4. qPCR

qPCR was performed as described in [12]. In brief, reactions were performed in triplicate with each reaction consisting of 5 µL of GoTaq^®^ qPCR Master Mix (Promega), 400 nM forward and reverse primers, 40 ng cDNA, and nuclease-free water (total volume = 10 µL). Primers for each innexin and our reference gene (ribosomal protein S7, RPS7) were used as in Calkins and Piermarini [12]; see Supplementary Table S1. The reactions were then subjected to the following thermocycling protocol using a C1000/CFX96 real time system (Bio-Rad Laboratories, Hercules, CA, USA): initial denaturation of 95 °C (3 min), followed by 39 cycles of 95 °C (10 s), and 58 °C (30 s), ending with a melt curve cycle. qPCR results were analyzed using the ΔCt method [18,19] and expressed as relative gene expression.

### 2.5. dsRNA Synthesis and Injections

dsRNA synthesis and injections were performed as described in Calkins and Piermarini [12]. In brief, primers for dsRNA template synthesis were designed to amplify 300–500 bp gene segments (Calkins and Piermarini [12]; see Supplementary Table S2) to be utilized in the T7 MEGAscript^®^ dsRNA synthesis kit (Thermo Fisher Scientific). dsRNA was synthesized following the manufacture’s protocols and stored at −80 °C.

On the day of an injection, dsRNA was diluted to 1 µg/µL in 0.5× PBS solution (5.95 mM phosphates, 68.5 mM sodium chloride, and 1.35 mM potassium chloride; pH 7.5; Fisher Scientific, Fairlawn, NJ, USA). Eighty mosquitoes were injected with 1 µg of either *inx2* dsRNA or *eGFP* dsRNA and returned to rearing conditions with access to 10% sucrose. After three days, three mosquitoes were removed from each treatment for knockdown assessment via qPCR and the remaining mosquitoes were utilized in fecundity assays (see Section 2.6). For tissue level knockdown analysis, 40 mosquitoes were injected with dsRNA for either *inx2* or *eGFP* and dissected three days post injection.

### 2.6. Fecundity and Viability Assays

Three days after dsRNA injection, mosquitoes were offered a blood meal (see Section 2.2); those with no visible blood in their abdomens were excluded from the assays. Twenty-four hours later, the blood-fed mosquitoes were transferred to individual egg-laying tubes for the fecundity assay. The egg-laying tubes consisted of a cylindrical glass tube (21 × 70 mm; Fisher Scientific, Pittsburg, PA, USA) with a piece of coffee filter (Melitta, Clearwater, FL, USA) cut to fit the bottom of the tube. The filter was wetted with 150 µL of ddH_2_O (Milli-Q^®^ filtered water, Merck KGaA, Darmstadt, Germany) and the open end was plugged with a cotton ball. The mosquitoes in their individual egg-laying tubes were returned to rearing conditions for 48 h, and the number of eggs laid by each mosquito was counted.

After counting the eggs, filter papers were consolidated according to dsRNA treatment and allowed to dry in a rearing chamber for one week. Eggs were then hatched in ddH_2_O under vacuum and returned to rearing conditions for 24 h. The resulting larvae were immobilized through refrigeration before counting them under a dissection stereomicroscope (World Precision Instruments, Sarasota, FL, USA/model PZMTIII-BS).

### 2.7. Data Analysis

All data were analyzed with GraphPad Prism 6 (GraphPad Software, La Jolla, CA, USA). Innexin mRNA expression levels between blood-fed and non-blood-fed treatments were analyzed via a two-way ANOVA with a Sidak post hoc analysis. The statistical significance of innexin mRNA knockdown after dsRNA injection was determined with *t*-tests comparing mosquitoes injected with eGF*p* and inx2 dsRNAs. Fecundity measurements (percent mosquitoes ovipositing, percent viability) were analyzed with one-way ANOVAs. Numbers of eggs laid were analyzed with a non-parametric Kruskal-Wallis ANOVA and Dunn’s post-test.

## 3. Results

### 3.1. mRNA Expression

**Figure 1:**
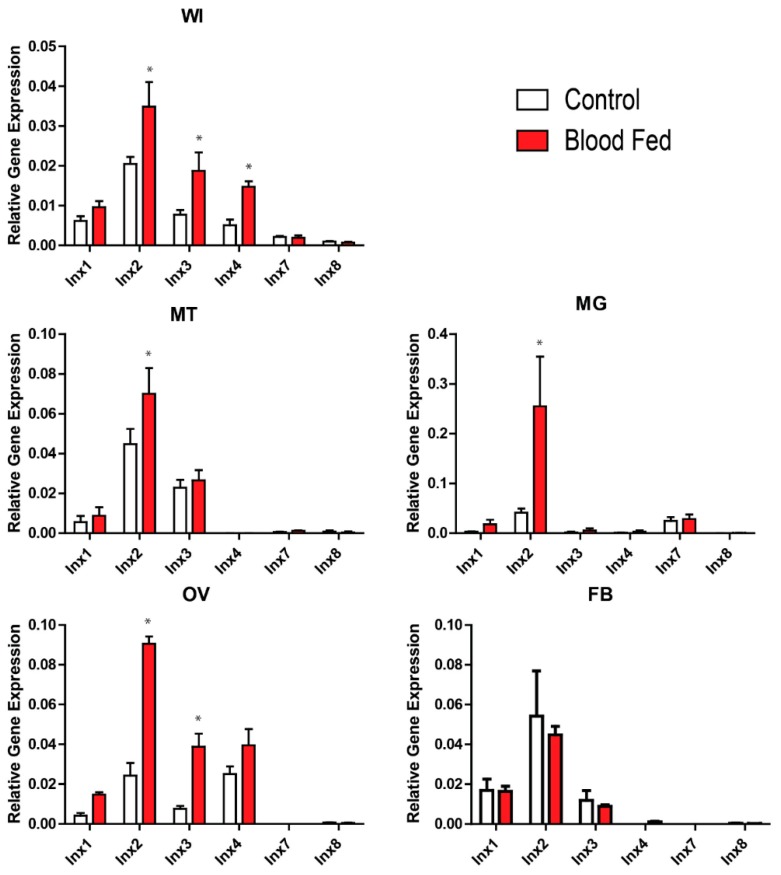
Effects of a blood meal on innexin mRNA expression 24 h post-blood meal. White bars indicate non-blood-fed control females and red bars indicate blood-fed females. Values are means ± SEM, *n* = 5. * indicate differences within a gene between blood-fed mosquitoes and non-blood-fed controls as determined by a two-way ANOVA and Newman-Keuls multiple comparison (*p* < 0.05). WI = whole insect; MT = Malpighian tubules; MG = midgut; OV = ovaries; FB = fat body.

At 3 h PBM, there were no significant differences in innexin mRNA levels between blood-fed and non-blood-fed mosquitoes (Supplementary Figure S1). However, at 24 h PBM, at least one innexin was differentially expressed between blood-fed and non-blood-fed mosquitoes at the whole mosquito and tissue levels, except in the fat body (Figure 1). At the whole mosquito level, *inx2*, *inx3*, and *inx4* were significantly upregulated. At the tissue level, *inx2* was significantly upregulated in the Malpighian tubules, midgut, and ovaries, and *inx3* was significantly upregulated in the ovaries.

### 3.2. RNAi

**Figure 2:**
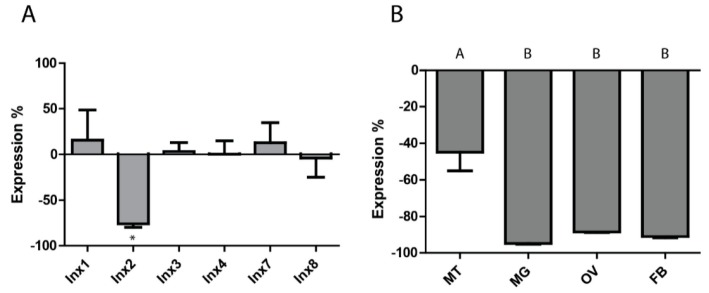
RNAi-induced knockdown of *inx2* mRNA expression. (**A**) Whole insect innexin mRNA expression three days post injection of *inx2* dsRNA (percent relative to eGF*p* dsRNA-injected controls). * indicates significant difference from *eGFP* dsRNA-injected mosquitoes as determined by a *t*-test (*p* < 0.05). Values are means ± SEM, *n* = 7; (**B**) Tissue-level innexin mRNA expression three days post injection of *inx2* dsRNA. Abbreviations are as in Figure 1. Values are means ± SEM, *n* = 3. Letters represent differences as determined by one-way ANOVA and Newman-Keuls multiple comparison (*p* < 0.05).

Given that *inx2* was upregulated in the whole insect, Malpighian tubules, midgut, and ovaries at 24 h PBM, it was selected for RNAi experiments. Injecting 1 µg of *inx2* dsRNA into adult females resulted in a 75.7 ± 3.9% reduction in mRNA expression (relative to controls injected with *eGFP* dsRNA) at the whole mosquito level within three days, with no significant changes in the expression levels of the other innexin mRNAs (Figure 2A). A similar degree of knockdown persisted 24 h after a blood meal (Supplementary Figure S2). At the tissue level, knockdown of *inx2* was similar in the midgut (94.8 ± 0.5%), ovaries (88.5 ± 0.2%), and fat body (91.0 ± 0.7%), while knockdown of *inx2* in the Malpighian tubules was significantly weaker (44.9 ± 10.1%) (Figure 2B).

**Figure 3:**
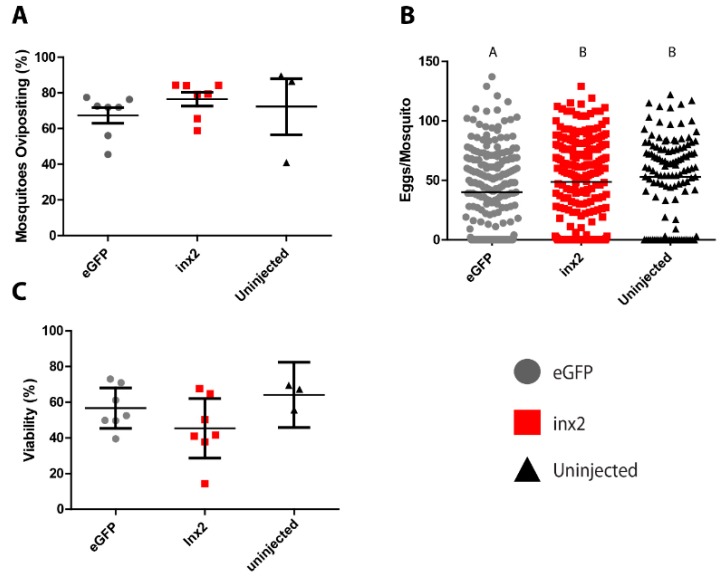
Effects of *inx2* knockdown on fecundity of adult female mosquitoes. (**A**) Effect of *inx2* knockdown on percentage of mosquitoes ovipositing. Values are means (horizontal black bars) ± SEM and individual data points are shown for each replicate (*n* = 7 for *eGFP*, 7 for *inx2*, and 3 for untreated); (**B**) Effects of *inx2* knockdown on number of eggs laid. Horizontal black bars indicate median number of eggs laid per mosquito, and individual data points represent individual mosquitoes (*n* = 231 for *eGFP*, 235 for *inx2*, and 119 for untreated). Letters represent differences as determined by a non-parametric one-way ANOVA and Dunn’s multiple comparison; (**C**) Effect of *inx2* knockdown on percentage of eggs hatching into viable larvae. Values are means (horizontal black bars) ± SEM and individual data points are shown for each replicate (*n* = 7 for *eGFP*, 7 for *inx2*, and 3 for untreated).

The injection of *inx2* dsRNA did not significantly affect the median number of eggs laid (55 eggs per mosquito) compared to uninjected controls (60 eggs per mosquito; Figure 3A). However, mosquitoes injected with *eGFP* dsRNA laid significantly fewer eggs (median = 40 eggs per mosquito) than both the *inx2* dsRNA-injected and uninjected mosquitoes (Figure 3A). No significant differences were found among the treatments in the viability of eggs (*eGFP* = 56.7 ± 4.6%; *inx2* = 45.4 ± −6.8%; uninjected = 64.1 ± 4.3%; Figure 3B) or the percentage of mosquitoes that oviposited (*eGFP* = 67.4 ± 4.5%, *inx2* = 76.5 ± 3.9%, uninjected = 72.3 ± 15.7%; Figure 3C).

## 4. Discussion

Our study is the first to quantify innexin mRNA expression in adult female *Ae. aegypti* following a blood meal. In particular, we found that *inx2* and *inx3* were upregulated by 24 h PBM in the whole insect, which can in part be attributed to an upregulation of *inx2* in the Malpighian tubules, midgut, and ovaries, and *inx3* in the ovaries (Figure 1). Although *inx4* was also upregulated by 24 h PBM in the whole insect, we did not find a corresponding upregulation in any of the tissues examined (Figure 1). Thus, the site of *inx4* upregulation remains to be determined.

After a blood meal, the midgut contributes to digestion, nutrient absorption, immune responses, and xenobiotic detoxification, while the Malpighian tubules contribute to diuresis as well as xenobiotic and metabolite detoxification/excretion [20,21,22,23]. Additionally, the ovaries undergo vitellogenesis and dramatically increase in size before eggs are oviposited [24,25]. Thus, these tissues undergo profound physiological changes after the mosquito ingests a blood meal. The consistent upregulation of *inx2* in all three of these tissues at 24 h PBM suggests that *inx2*-mediated intercellular communication may contribute to the regulation of the physiological activities in these tissues. Alternatively, *inx2* could be acting as a hemichannel in the plasma membranes of these tissues to release signaling molecules that contribute to paracrine/endocrine communication after a blood meal, as suggested by Li et al. [8] for *inx1* in the midgut of *An. gambiae*.

As such, we hypothesized that knockdown of *inx2* would disrupt digestion, excretion, and/or oogenesis after a blood meal, thereby resulting in a reduction of fecundity. However, despite knocking down *inx2* mRNA levels by ~75% in whole mosquitoes, ~45% in the Malpighian tubules, and ~90% in midgut and ovaries (Figure 2), we found no reduction in the percentage of mosquitoes that oviposited, the number of eggs laid per mosquito, or the viability of the eggs in *inx2* dsRNA-injected females compared to uninjected mosquitoes (Figure 3). Although these results suggest that *inx2* does not contribute to fecundity, we cannot rule out that inx2 protein levels were unaffected by the mRNA knockdown, thereby resulting in no detectable phenotype. If this were the case, potential contributing factors could be slow turnover rates of inx2 proteins and functional redundancy among the innexins. Innexin turnover rates are not currently known, but mRNA expression analyses of innexins in several mosquito tissues suggest that the molecular potential for functional redundancy exists [11]. Intriguingly, *eGFP* dsRNA-injected mosquitoes laid fewer eggs than the *inx2* dsRNA-injected and uninjected control mosquitoes (Figure 3), suggesting a potential consequence of *eGFP* dsRNA injection on fecundity. In the honeybee *Apis mellifera*, injection of *GFP* dsRNA results in differential expression of ~10% of the insect’s transcriptome, including an upregulation of immune genes [26]. Thus, it is possible that injection of *eGFP* dsRNA in *Ae. aegypti* elicits a similar response that could potentially divert energetic resources from oogenesis.

## 5. Conclusions

The present study is the first to quantify the effects of a blood meal on the molecular expression of innexins in *Ae. aegypti*. While we found *inx2* to be highly upregulated in both the midgut and ovaries following a blood meal, RNAi of *inx2* yielded no reduction in fecundity. Taken together, the results from this study provide molecular evidence that innexins are potentially important in the physiology of *Ae. aegypti* after a blood meal, but their specific functional roles remain to be elucidated.

## Supplementary Materials

The following are available online at www.mdpi.com/2075-4450/8/4/122/s1, Table S1: qPCR primer pairs. Each set of innexin primers was determined to be specific through melt curve analysis and DNA sequencing of PCR products, Table S2: dsRNA template synthesis primers. Each primer set consists of an innexin specific region for amplification of the target gene from plasmid, and the T7 promoter sequence (TAATACGACTCACTATAGGGAGA), Figure S1: Effects of a blood meal on innexin mRNA expression 3-h post-blood meal, Figure S2: Knockdown of inx2 in blood-fed and non-blood-fed mosquitoes.
