# Supplementary material for: A Blood Meal Enhances Innexin mRNA Expression in the Midgut, Malpighian Tubules, and Ovaries of the Yellow Fever Mosquito Aedes aegypti
Source: Insects. 2017 Nov 6;8(4):122. doi: 10.3390/insects8040122 (PMC5746805; doi:10.3390/insects8040122)
Supplement: Supplementary file 1 [file insects-08-00122-s001.pdf]

insects

MDPI

Communication

A Blood Meal Enhances Innexin mRNA Expression in the Midgut, Malpighian Tubules, and Ovaries of the Yellow Fever Mosquito *Aedes aegypti*

Travis L. Calkins and Peter M. Piermarini *

Supplemental Tables and Figures:

Supplemental Table S1. qPCR primer pairs. Each set of innexin primers was determined to be specific through melt curve analysis and DNA sequencing of PCR products.

	qPCR Forward	qPCR Reverse
**Inx1**	CACCGATAGTGCCGTATTCC	CCGACATATTGTGTGGCAGT
**Inx2**	GGAGATCCTATGGCACGAGT	ACGGTAGCACACAGAGTCCA
**Inx3**	TCGTTCGGTTACTTCATCTGC	GCGATTCTCCTGATCCATGTC
**Inx4**	TTCTGTTGGACACTGGGAAC	CCATGTGCGTTCCTATTTCG
**Inx7**	TGGGTCCCGTTTGTGTTATT	CCATACGAAGACCATCCACA
**Inx8**	GACTGCGTTCACACGAAAGA	GGGTACTTCGCTACCGACTTT
**RPS7**	CTTTGATGTGCGAGTGAACAC	CATCTCCAACTCCAGGATAGC

Supplemental Table S2. dsRNA template synthesis primers. Each primer set consists of an innexin specific region for amplification of the target gene from plasmid, and the T7 promoter sequence (TAATACGACTCACTATAGGGAGA).

	dsRNA Template Forward	dsRNA Template Reverse
**Inx2**	TAATACGACTCACTATAGGGAGATTT GGCGTTTGAAAAGTGTG	TAATACGACTCACTATAGGGAGAATACTC CCGGCTGAGCAATA
**eGFP**	TAATACGACTCACTATAGGGACGTAA ACGGCCACAAGTT	TAATACGACTCACTATAGGGTTGGGGTCT TTGCTCAGG

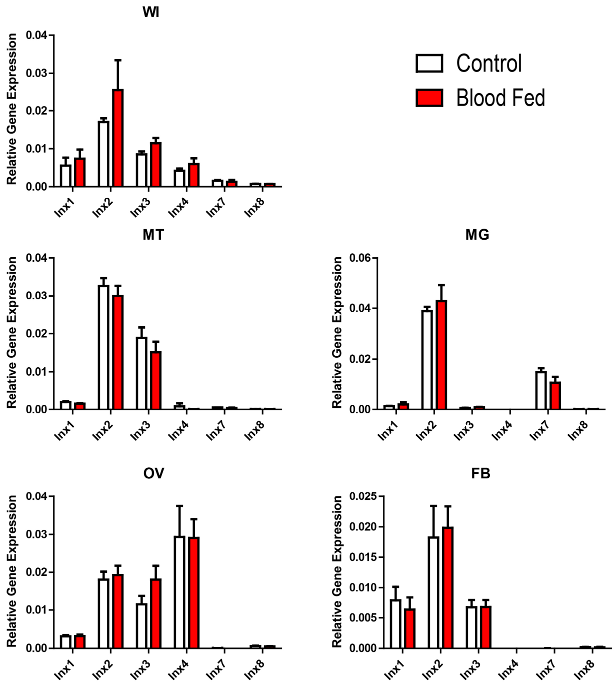

Supplemental Figure S1. Effects of a blood meal on innexin mRNA expression 3-h post-blood meal. White bars indicate non-blood fed control females and red bars indicate blood fed females. Bars indicate means ± SEM, *n* =5. Abbreviations are as in Figure 1. No differences were found in innexin expression between blood fed and non-blood fed controls as determined by a two-way ANOVA.

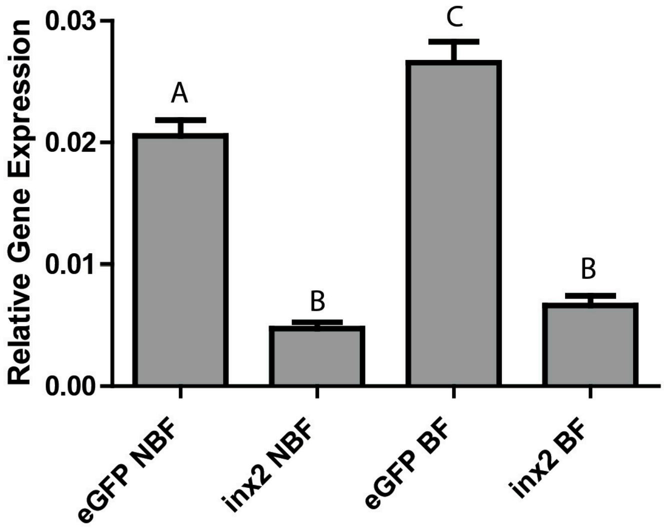

Supplemental Figure S2. Knockdown of *inx2* in blood fed and non-blood fed mosquitoes. Bars indicate means ± SEM, *n* = 3. Letters represent statistical differences as determined by a two-way ANOVA. Knockdown of *inx2* is not significantly different between blood-fed and non-blood-fed mosquitoes.
